# Mobile Robot Navigation with Enhanced 2D Mapping and Multi-Sensor Fusion

**DOI:** 10.3390/s25082408

**Published:** 2025-04-10

**Authors:** Basheer Al-Tawil, Adem Candemir, Magnus Jung, Ayoub Al-Hamadi

**Affiliations:** Neuro-Information Technology, Otto-von-Guericke-University Magdeburg, 39106 Magdeburg, Germany; ademcandemir1@gmail.com (A.C.); magnus.jung@ovgu.de (M.J.)

**Keywords:** SLAM, localization gmapping algorithm, navigation, data fusion, point cloud

## Abstract

This paper presents an enhanced Simultaneous Localization and Mapping (SLAM) framework for mobile robot navigation. It integrates RGB-D cameras and 2D LiDAR sensors to improve both mapping accuracy and localization efficiency. We propose a data fusion strategy where RGB-D point clouds are projected into 2D and denoised alongside LiDAR data. Late fusion is applied to combine the processed data, making it ready for use in the SLAM system. Additionally, we propose the enhanced Gmapping (EGM) algorithm by adding adaptive resampling and degeneracy handling to address particle depletion issues, thereby improving the robustness of the localization process. The system is evaluated through simulations and a small-scale real-world implementation using a Tiago robot. In simulations, the system was tested in environments of varying complexity and compared against state-of-the-art methods such as RTAB-Map SLAM and our EGM. Results show general improvements in navigation compared to state-of-the-art approaches: in simulation, an 8% reduction in traveled distance, a 13% reduction in processing time, and a 15% improvement in goal completion. In small-scale real-world tests, the EGM showed slight improvements over the classical GM method: a 3% reduction in traveled distance and a 9% decrease in execution time.

## 1. Introduction

In recent years, mobile robots have become essential across various industries and are now a big part of our daily lives [1,2,3]. As these robots take on more complex tasks, their ability to navigate and understand their surroundings becomes crucial [1,2]. SLAM is a fundamental technique in robotics and autonomous systems, enabling them to construct a map of an unknown environment while simultaneously determining their own position within it [4,5]. It achieves this by processing sensor data from cameras, LiDAR, or sonar, combined with advanced algorithms such as Kalman filters, particle filters, and graph-based optimization [6,7]. It is widely used in applications like mobile robots, virtual reality (VR), augmented reality (AR), and self-driving cars [6,7,8].

As research in SLAM advances, improving its efficiency and accuracy remains a key focus to enhance autonomous navigation in real-world scenarios [9]. A major challenge is balancing high accuracy with computational efficiency, especially in environments with dynamic obstacles, low-feature areas, or changing lighting conditions [7]. SLAM methods are generally divided into two categories: feature -based methods [10] and direct methods [11]. Feature-based approaches, such as ORB-SLAM [12] and PTAM (Parallel Tracking and Mapping) [13], rely on detecting and tracking key points across frames, which can be computationally demanding in textureless environments. Direct methods, like LSD-SLAM [14] and DSO [15], use the raw brightness of images, so they are more affected by light changes or blurry motion.

Many SLAM methods rely on high-precision sensors, such as 3D LiDAR, which provide detailed spatial data but are expensive and computationally intensive [16,17]. Vision-based SLAM, using RGB or RGB-D cameras, offers a more affordable solution but faces challenges, especially in dynamic environments and feature-poor areas [5,18]. Additionally, relying on a single sensor type limits performance in complex environments, as each sensor has its own limitations, such as poor accuracy in low-light conditions or difficulty in capturing fine details in featureless areas [19]. To overcome these challenges, SLAM methods need to integrate multiple sensor modalities to improve robustness and accuracy [20].

One solution to these challenges is the use of hybrid approaches that combine the strengths of different sensors [21,22]. Multi-sensor SLAM, which integrates sensors like LiDARs and RGB-D cameras, has gained significant attention in recent years [23]. By fusing the spatial precision of LiDAR with the rich visual context provided by RGB-D cameras, these approaches can handle dynamic and feature-sparse environments more effectively [19]. This fusion approach leverages data from various sensors. It employs information fusion algorithms to improve pose estimation, trajectory estimation, and mapping. As a result, robots gain enhanced environmental awareness and better navigation in complex scenarios [23,24]. To reduce computational complexity, RGB-D camera data can be converted into 2D laser scans. This is performed by projecting 3D point clouds, making real-time navigation possible in resource-constrained environments [21,25]. Furthermore, traditional SLAM algorithms like Gmapping rely on particle filters. Their performance can be improved by refining how particles are handled. Combining this with sensor information leads to better overall results [23,26].

**Figure 1:**
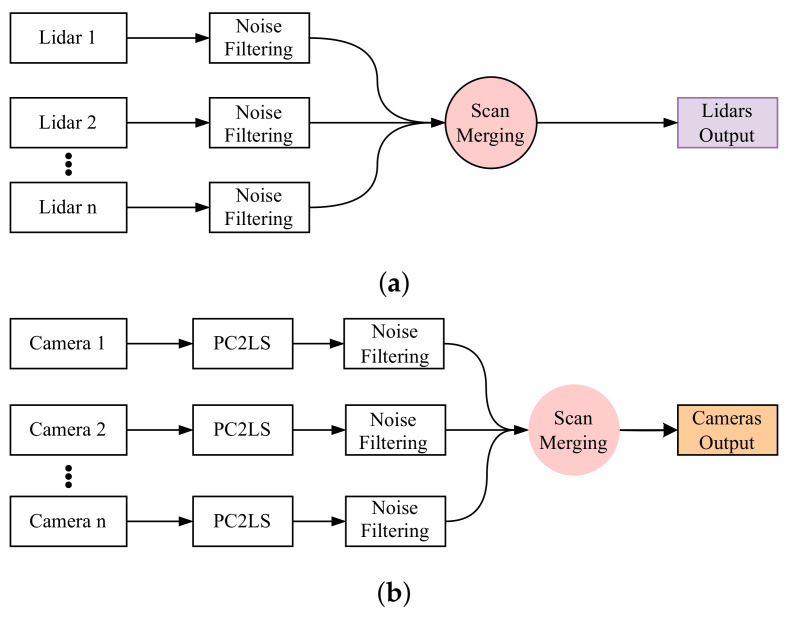
Fusion of LiDAR and camera data, including noise filtering and converting camera point clouds to laser scans (PC2LS), preparing the data for robot navigation. (**a**) LiDAR fusion and (**b**) camera fusion.

**Figure 2:**
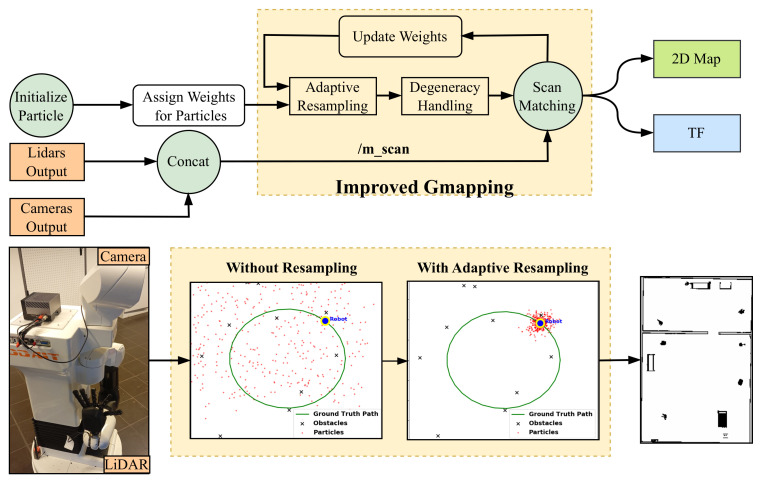
The process of combining LiDAR and camera outputs, as shown in Figure 1a,b, to generate a 2D map. This process includes preparing the m_scan topic from sensor fusion, initializing the particles and assigning them weights, performing scan matching between sensors and particles to update the weights, and refining the estimation. Additionally, adaptive resampling is applied, and degeneracy is handled within the mapping algorithm. The final output is a 2D map, transformation tf.

In summary, the contributions of our work can be listed as follows:

- Point Cloud Projection Broadcasting: We implemented a system for projecting RGB-D camera point clouds as 2D laser scans. This involves preparing the data for seamless integration with other sensors by utilizing transformation matrices and leveraging existing ROS packages; see Figure 1b.
- Modular Multi-Sensor Fusion with Noise Filtering: The system incorporates multiple sensors in parallel with early fusion, designed for adaptability and modularity. If one sensor fails, the system can continue functioning. Additionally, a noise filtering block is added after each sensor to improve data quality and reliability. See Figure 1a,b.
- Enhanced Gmapping with Adaptive Resampling: We improved Gmapping by adding adaptive resampling and degeneracy handling. Resampling occurs only when needed, ensuring a well-balanced particle distribution with proper weights (see Figure 2).

**Figure 3:**
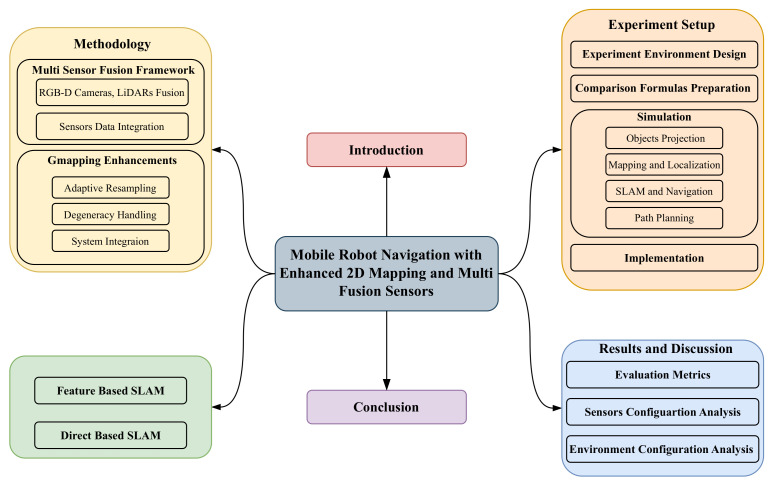
This paper’s organizational chart.

As shown in Figure 3, the paper is divided into six main sections. Section 1 provides an introduction to 2D mapping, outlining its challenges and proposed solutions. Section 2 reviews related work, offering a detailed analysis of existing methodologies and their limitations. Section 3 explains the methodology, covering data acquisition, point cloud processing, sensor vision integration, and data broadcasting. Section 4 focuses on the experiments, describing the simulation environment, object projection, performance evaluation metrics, and real-world implementation in a small-scale area. Section 5 discusses the results, examining sensor configurations, environmental setups, and system performance while highlighting their practical implications. Finally, Section 6 concludes the paper by summarizing the key contributions, insights, and potential avenues for future research.

## 2. Related Work

SLAM has seen significant advancements, leveraging diverse sensors such as LiDAR, RGB-D cameras, and inertial measurement units (IMUs) to enable robots to navigate and create accurate maps of their environments [8,18,27,28]. The data from these sensors allow for constructing maps in multiple formats, ranging from simple 2D grids to complex 3D semantic and topological maps, each suited to different application needs [29,30,31]. Among these, 2D grid maps are often preferred for their computational simplicity and suitability for real-time navigation tasks [32].

SLAM methods are usually categorized into two main approaches: feature -based methods and direct methods. Feature-based approaches extract and track keypoints across frames, making them suitable for structured environments [4,33,34]. Direct methods, on the other hand, operate on raw image intensities, providing robustness in low-texture regions but demanding higher computational resources. Additionally, SLAM can be enhanced through multi-sensor fusion techniques, which integrate multiple data sources to improve accuracy and robustness [35,36,37]. This section provides an overview of these approaches and their challenges.

### 2.1. Feature-Based SLAM Approaches

Feature-based SLAM methods extract and match keypoints to estimate motion and build a map. These approaches include ORB-SLAM [12,31], PTAM [13], RTAB-Map [38], and Gmapping [20], each offering distinct advantages.

ORB-SLAM: It is a feature-based SLAM system that employs ORB descriptors for keypoint detection and matching, enabling real-time performance through a three-threaded architecture: tracking, local mapping, and loop closing [31]. Camera pose estimation is achieved by minimizing the re-projection error via Bundle Adjustment (BA):

(1) $$E=\sum_{i}{\left|x_{i}-\pi \left(P,X_{i}\right)\right|}^{2}$$

where $x_{i}$ represents the observed image coordinates, $X_{i}$ is the 3D world point, and $\pi (P,X_{i})$ is the projection function given the camera pose P. ORB-SLAM further refines global consistency through pose graph optimization over the Essential Graph. However, ORB-SLAM struggles in low-texture environments, where few keypoints can be detected, and dynamic scenes, where moving objects cause tracking errors.

PTAM:

It introduced a dual-thread approach, separating tracking and mapping with bundle adjustment background. It is designed to improve real-time performance in small-scale environments [30]. PTAM optimizes the 3D map and camera poses using bundle adjustment, minimizing the re-projection error:

(2) $$\arg\min_{X,C}\sum_{i,j}w_{ij}{∥p_{ij}-K\left[R_{j}|t_{j}\right]X_{i}∥}^{2}$$

where $X_{i}$ is the 3D point, $C_{j}=\left(R_{j},t_{j}\right)$ is the camera pose, $p_{ij}$ is the observed 2D projection, and *K* is the intrinsic camera matrix. The weight $w_{ij}$ adjusts measurement reliability. While PTAM ensures local accuracy, its lack of loop closure limits long-term consistency, leading to accumulated drift over long trajectories.

RTAB-Map:

RTAB-Map (Real-Time Appearance-Based Mapping) is often classified as a feature-based SLAM method that enhances mapping consistency by integrating loop closure detection. It incorporates a memory management strategy, enabling long-term online operation by discarding redundant keyframes while preserving mapping accuracy [34].

The graph optimization in RTAB-Map is typically performed using pose graph optimization, expressed as:

$$E\left(T\right)=\sum_{(i,j)\in E}{\left|f\left(T_{i},T_{j}\right)-z_{ij}\right|}_{\Omega_{ij}}^{2}$$

where $T_{i}$ and $T_{j}$ are the transformation matrices for keyframe *i* and keyframe *j*, $z_{ij}$ represents the relative pose measurement between keyframe *i* and keyframe *j*, and $\Omega_{ij}$ is the information matrix, which encodes the uncertainty of the relative pose measurement. RTAB-Map is particularly effective for large-scale environments but comes with high computational demands, which may pose challenges for deployment on resource-constrained robotic platforms [32].

Gmapping: Gmapping is commonly categorized as a feature-based, particle filter-based SLAM algorithm that utilizes Rao–Blackwellized Particle Filters (RBPFs) to estimate robot trajectories while building a 2D occupancy grid map [32]. The posterior probability of the map *m* and trajectory $x_{1:t}$ given the observations $z_{1:t}$ and controls $u_{1:t}$ is:

(3) $$P\left(x_{1:t},m|z_{1:t},u_{1:t}\right)=P\left(m|x_{1:t},z_{1:t}\right)P\left(x_{1:t}|z_{1:t},u_{1:t}\right)$$

where the first term represents mapping given known poses, and the second term represents localization.

It is widely used for indoor navigation due to its computational efficiency. However, Gmapping suffers from particle degeneracy, where low-weight particles accumulate over time, reducing localization accuracy. Furthermore, it struggles in large-scale environments due to its reliance on odometry, which introduces cumulative errors [26].

### 2.2. Direct SLAM Approaches

Unlike feature-based methods, direct SLAM approaches estimate motion by optimizing pixel intensity values directly, eliminating the need for explicit feature extraction. Notable direct methods include LSD-SLAM [14] and DSO (Direct Sparse Odometry) [15].

LSD-SLAM: LSD-SLAM reconstructs semi-dense depth maps using image intensity variations rather than discrete keypoints [14]. It relies on photometric error minimization:

(4) $$E=\sum_{i}{\left(I_{i}-I\left(T\cdot X_{i}\right)\right)}^{2}$$

where $I_{i}$ is the image intensity at pixel *i*, and T represents the estimated camera pose. This makes LSD-SLAM effective in low-texture environments, where feature-based methods typically fail. However, it suffers from high computational costs and is highly sensitive to photometric distortions such as lighting changes.

DSO:

Direct Sparse Odometry (DSO) improves upon LSD-SLAM by optimizing a sparse subset of pixels, significantly reducing computational requirements while maintaining accurate motion estimation [15]. However, it does not incorporate loop closure detection, making it prone to drift over long trajectories.

Feature-based SLAM is effective in structured environments but struggles in low-texture areas where keypoints are hard to detect, leading to tracking failures. It also has high computational costs due to real-time feature extraction [39,40]. For example, Gmapping suffers from particle degeneracy, reducing accuracy and increasing computational load, while RTAB-Map requires high memory for loop closure detection [41]. Direct SLAM, on the other hand, performs well in feature-poor environments but has its own challenges. It requires high computational power to process raw pixel intensities in real time [22]. The absence of loop closure detection causes drift and reduces global consistency, while sensitivity to lighting changes and reflections affects accuracy [42].

In conclusion, feature-based SLAM ensures better global consistency but struggles in textureless environments and requires high computational resources [41]. Also, direct SLAM is robust in feature-poor areas but lacks loop closure detection and is sensitive to lighting variations [22]. To address these challenges, researchers have developed a hybrid SLAM, combining feature-based tracking with direct methods to handle feature sparsity [20,43,44]. Multi-sensor fusion, integrating LiDAR, cameras, and IMUs, improves robustness by compensating for missing visual features [39,40].

## 3. Methodology

This section elaborates on the improved Gmapping algorithm, a SLAM framework that fuses data from 2D LiDAR and RGB-D cameras. Figure 2 illustrates the proposed framework, which integrates multi-sensor fusion with Gmapping enhancements, as explained in the following sections.

### 3.1. Multi Sensor Fusion Framework

The proposed approach combines data from multiple RGB-D cameras and 2D LiDAR sensors to create a unified and comprehensive environmental representation. RGB-D cameras capture both color and depth information, generating 3D point cloud data. These data points are projected onto a 2D plane to simplify processing while preserving critical spatial details. This projection reduces computational complexity while retaining depth information essential for navigation [21,39].

The methodology was adjusted to be able to accept multiple different sensors so that it includes 2D LiDAR sensors and RGB-D cameras to cover all around the robot environment and 360-degree field of view.

#### 3.1.1. RGB-D Camera and LiDAR Fusion

As shown in Figure 1b, the raw point cloud data generated by the RGB-D cameras is represented as n×3 matrices, where each point is defined by its coordinates (x,y,z), corresponding to the horizontal position, depth, and height, respectively. In our simulation case, the point cloud data from the front, back, left, and right cameras are represented as $F_{C},B_{C},L_{C},R_{C}$. To simplify the mapping and localization process, the 3D point cloud data are projected onto a laser scan (PC2LS), where the *z*-coordinate (height) is eliminated and the points are transformed into (x,y) coordinates. This transformation enables more efficient processing while maintaining essential depth information for navigation.

The projection process is mathematically represented as:

(5) $$\begin{array}{cc} F_{C} & =\left[\begin{array}{ccc} x_{fc1} & y_{fc1} & z_{fc1}\\ \vdots & \vdots & \vdots \\ x_{fcn} & y_{fcn} & z_{fcn} \end{array}\right],\;B_{C}=\left[\begin{array}{ccc} x_{bc1} & y_{bc1} & z_{bc1}\\ \vdots & \vdots & \vdots \\ x_{bcn} & y_{bcn} & z_{bcn} \end{array}\right]\\ L_{C} & =\left[\begin{array}{ccc} x_{lc1} & y_{lc1} & z_{lc1}\\ \vdots & \vdots & \vdots \\ x_{lcn} & y_{lcn} & z_{lcn} \end{array}\right],\;R_{C}=\left[\begin{array}{ccc} x_{rc1} & y_{rc1} & z_{rc1}\\ \vdots & \vdots & \vdots \\ x_{rcn} & y_{rcn} & z_{rcn} \end{array}\right] \end{array}$$

The resulting 2D representation, denoted as $F_{L}^{2D},B_{L}^{2D},F_{C}^{2D},B_{C}^{2D},L_{C}^{2D},R_{C}^{2D}$, contains the spatial coordinates in the (x,y) plane.

(6) $$\begin{array}{cc} F_{L}^{2D} & =\left[\begin{array}{cc} x_{f1} & y_{f1}\\ \vdots & \vdots \\ x_{fn} & y_{fn} \end{array}\right],\;B_{L}^{2D}=\left[\begin{array}{cc} x_{b1} & y_{b1}\\ \vdots & \vdots \\ x_{bn} & y_{bn} \end{array}\right]\\ F_{C}^{2D} & =\left[\begin{array}{cc} x_{fc1} & y_{fc1}\\ \vdots & \vdots \\ x_{fcn} & y_{fcn} \end{array}\right],\;B_{C}^{2D}=\left[\begin{array}{cc} x_{bc1} & y_{bc1}\\ \vdots & \vdots \\ x_{bcn} & y_{bcn} \end{array}\right]\\ L_{C}^{2D} & =\left[\begin{array}{cc} x_{lc1} & y_{lc1}\\ \vdots & \vdots \\ x_{lcn} & y_{lcn} \end{array}\right],\;R_{C}^{2D}=\left[\begin{array}{cc} x_{rc1} & y_{rc1}\\ \vdots & \vdots \\ x_{rcn} & y_{rcn} \end{array}\right] \end{array}$$

This information will be homogeneous and identical to the data coming from the LiDAR sensor; see Figure 1a.

**Figure 4:**
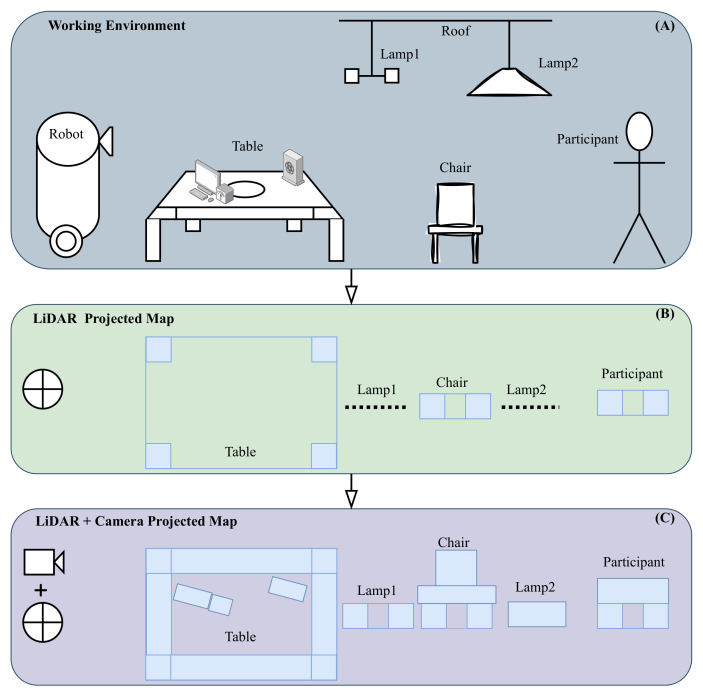
Illustration of an example working environment and the projected map of 3D objects in a 2D representation using LiDAR data compared to a combination of LiDAR and camera data. (**A**) Shows the actual 3D objects in the environment. (**B**) Shows the 2D projection of objects using only LiDAR sensor data. (**C**) Shows the 2D projection using combined LiDAR and camera data.

To demonstrate the effectiveness of the projection method, we set up the environment as shown in Figure 4. Rectangle A: Displays the full working environment, including a table, chairs, lamps, and computers, showing how the projection captures the scene. Rectangle B: Shows only the LiDAR data. It detects table legs and parts of the chair but does not capture elevated objects like lamps, illustrating the limitations of LiDAR-only perception. Rectangle C: Combines the LiDAR sensors with RGB-D cameras. Here, the boundaries of the table, the human body, and elevated objects like lamps are detected, demonstrating the enhanced ability to navigate and interact with the environment when both sensor types are used. This comparison highlights how combining LiDAR with RGB-D cameras provides a more covered view, improving the robot’s ability to navigate smoothly in environments and interact with objects at different heights.

#### 3.1.2. Sensor Data Integration

As shown in Figure 1a,b, after obtaining information from LiDAR sensors and RGB-D cameras, a filtration process is applied to extract accurate and clean data necessary for the overall system. The filtering process is performed at an early stage and on each individual sensor separately to improve processing speed and ensure system robustness, meaning the system can continue operating even if one sensor fails.

**Figure 5:**
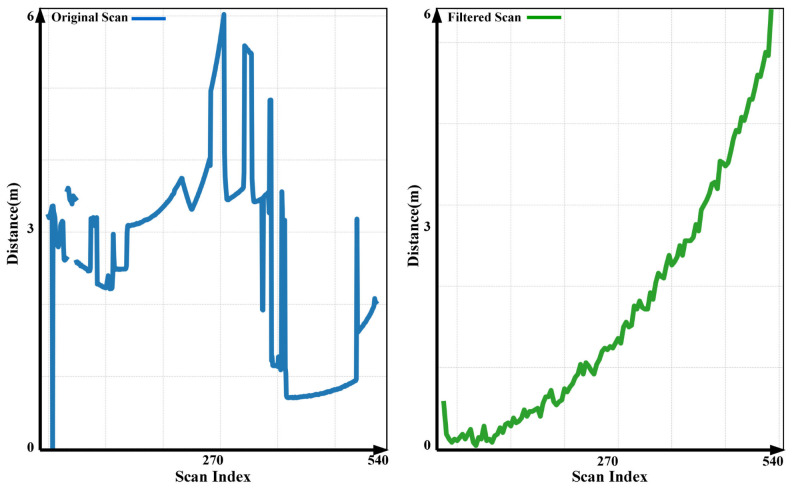
Comparison of raw and filtered LiDAR scans. The raw scan (**left**) contains noise, while the filtered scan (**right**) shows smoother and more stable readings (0–6 m). Each scan consists of up to 540 measurements.

To smooth the sensor data and reduce noise, we apply the moving average filter, which is computationally simple and effective for real-time applications [45,46]. The moving average filter is defined as follows:

(7) $$y\left[i\right]=\frac{1}{M}\sum_{j=0}^{M-1}x\left[i-j\right]$$

where y[i] is the filtered output at index *i*, *M* is the window size (number of previous samples used for averaging), and x[i−j] represents the raw sensor measurements. The effectiveness of this filtering technique in reducing noise from sensor readings is illustrated in Figure 5. The sensor data consist of up to 540 measurement points per scan, ranging between 0 and 6 m. The left side of the figure shows the raw sensor signal, while the right side presents the filtered output. The moving average filter effectively smooths fluctuations, removes unnecessary variations, and enhances data reliability for system integration.

After filtering and preparing the sensor data, each sensor publishes its information as an ROS topic with a unique name. To merge the filtered data, we utilize an existing ROS package [47], which combines multiple single-plane laser scans and 2D projections into a unified scan. The result is a 360-degree coverage, which is necessary for implementing SLAM methodologies.

The fused data are represented as:

(8) $$P_{\mathrm{merged}}=F_{L}^{2D}\cup B_{L}^{2D}\cup F_{C}^{2D}\cup B_{C}^{2D}\cup L_{C}^{2D}\cup R_{C}^{2D}.$$

where $F_{L}^{2D}$ and $B_{L}^{2D}$ represent the 2D projections from the front and back LiDAR sensors, and $F_{C}^{2D},B_{C}^{2D},L_{C}^{2D},R_{C}^{2D}$ correspond to the 2D projections from the RGB-D cameras at the front, back, left, and right. This merged data provide a comprehensive environmental map essential for real-time navigation and obstacle avoidance. Once the data are processed and fused, they are converted into a laser scan message, which is broadcast over the ROS firmware, making it accessible for use anywhere. Each point in the 2D matrix corresponds to a detected obstacle, with (x,y) coordinates indicating the position of the obstacle in the environment.

### 3.2. Gmapping Enhancements

**Figure 6:**
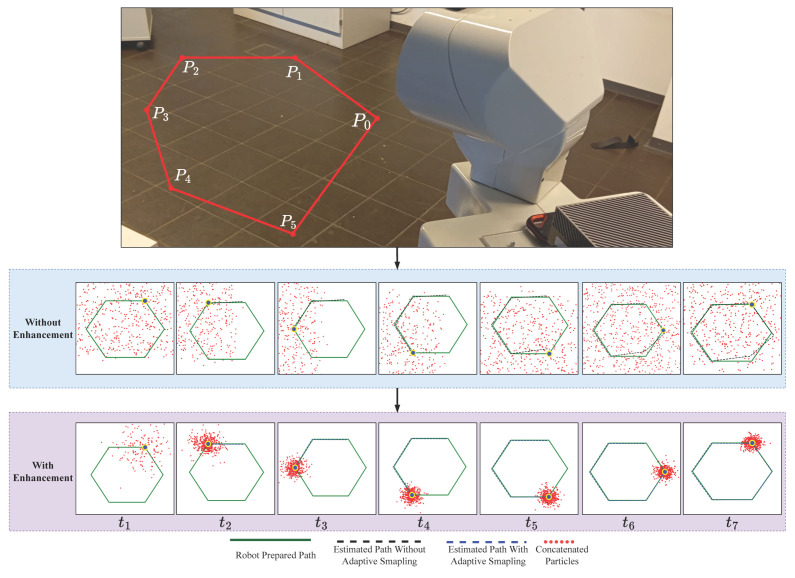
Effectiveness of adaptive resampling and degeneracy handling on the particle collection published by the sensors used in the robot. The time steps $t_{1},t_{2},\ldots ,t_{5}$ demonstrate the robot’s movement along the predefined path inside the environment. The figure shows the overlap of the estimated path and the given path in both cases by adding EGM and classical GM.

Gmapping is a widely used SLAM algorithm based on Rao–Blackwellized Particle Filters (RBPFs) [32]. While efficient, traditional Gmapping suffers from issues such as particle degeneracy, fixed grid resolution, and suboptimal scan matching [48,49]. In this section, we introduce simple enhancements to address these issues, see Algorithm 1 for the modified procedure. These changes result in slightly improved performance, as shown in Figure 6:

#### 3.2.1. Adaptive Resampling

Gmapping resamples particles at every step, which can lead to particle depletion [26,32]. To mitigate this, we introduce an adaptive resampling strategy based on the Effective Sample Size (ESS), defined as:

(9) $$N_{eff}=\frac{1}{\sum_{i=1}^{N}{\left(w_{t}^{i}\right)}^{2}}$$

where $N_{eff}$ represents the number of effectively independent particles, and $w_{t}^{i}$ is the weight of the *i*-th particle at time *t*. Resampling is performed only where $N_{eff}$ is within the threshold $N_{th}$. The threshold can be decided based on the size of the environment where the robot is working.

The resampling process is as follows:

1. Compute the cumulative sum of particle weights:
  $$C_{i}=\sum_{j=1}^{i}w_{j}$$
2. Generate *N* random values from a uniform distribution U∈[0,1].
3. Select a particle for each random value based on:

$$i=\min\{k:C_{k}>U_{j}\},\;j=1,\ldots ,N$$

where *i* is the index of the selected particle, *k* is the index in the cumulative sum $C_{k}$, and $C_{k}$ is the cumulative weight sum up to the *k*-th particle. Then, use these selected particles to prepare the map.

#### 3.2.2. Degeneracy Handling

Particle degeneracy occurs when only a few particles retain significant weights, leading to poor state estimation [49]. To address this, particle weights are normalized as follows:

(10) $$w_{t}^{i}=\frac{w_{t}^{i}}{\sum_{j}w_{t}^{j}}$$

Additionally, noise injection is applied by perturbing low-weight particles with small random displacements. This approach enhances particle spread, improving robustness against localization errors and preventing particle depletion.

The EGM algorithm integrates the proposed improvements which are shown in the following:
**Algorithm 1** Enhanced Gmapping Algorithm  1:Initialize particle set $P_{0}$ and initial map  2:**for** each timestep *t* **do**  3:   Predict particle states $x_{t}^{\left(i\right)}$ using the motion model  4:   Apply scan matching to refine $x_{t}^{\left(i\right)}$ using ICP  5:   Update particle weights $w_{t}^{\left(i\right)}$ based on sensor observations  6:   Compute $N_{eff}=\frac{1}{\sum_{i=1}^{N}{\left(w_{t}^{\left(i\right)}\right)}^{2}}$  7:   **if** $N_{eff}<N_{th}$ **then**  8:     Perform systematic resampling to maintain diversity  9:   **end if**10:   Normalize particle weights: $w_{t}^{\left(i\right)}=\frac{w_{t}^{\left(i\right)}}{\sum_{j}w_{t}^{\left(j\right)}}$11:   Inject small noise into low-weight particles to prevent degeneration12:   Output estimated pose ${\hat{x}}_{t}$ and map ${\hat{m}}_{t}$13:**end for**

#### 3.2.3. System Integration

**Figure 7:**
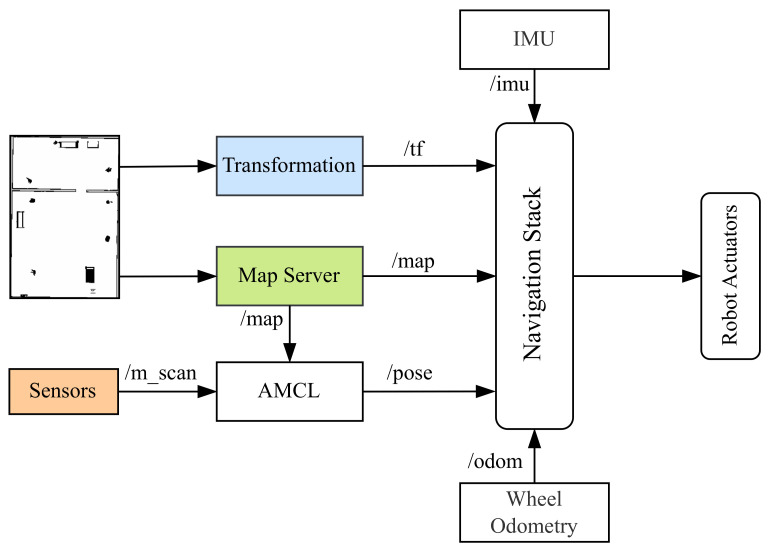
Navigation stack integrating multi-sensor fusion and enhanced Gmapping. The system receives sensor data from LiDAR, RGB-D cameras stored in (/m_scan), IMU, and odometry, which are processed and transformed (/tf) into a consistent representation. The enhanced Gmapping algorithm updates the occupancy grid map (/map), while AMCL utilizes the sensor data and transformations for precise localization.

As a final step, we combine all sensor fusions, enhanced Gmapping, and the already prepared navigation stack. The navigation stack is responsible for path planning, localization, and obstacle avoidance, ensuring smooth robot navigation. As shown in Figure 7, it consists of key components such as the map server, which provides an updated grid map, and AMCL, which estimates the robot’s position using sensor data. The transformation module (/tf) manages coordinate frames, while various sensors, including LiDAR, RGB-D cameras, IMU, and odometry, provide real-time data for accurate navigation. The multi-sensor fusion framework integrates data from these sources and converts it into a laser scan (/m_scan), which is then used by AMCL to improve localization. Additionally, the EGM refines the occupancy grid map (/map) by improving scan matching and particle filtering, ensuring better accuracy in dynamic environments. Together, these components enable the robot to build a precise map, estimate its position reliably, and navigate efficiently in complex surroundings.

## 4. Experiment Setup

The proposed SLAM system was evaluated using the Tiago robot. The setup featured front and back LiDAR sensors and cameras at four positions (front, back, left, and right), providing 360-degree coverage. This configuration enabled the robot to detect obstacles, navigate effectively, and interact with its surroundings.

### 4.1. Experiment Environment Design

**Figure 8:**
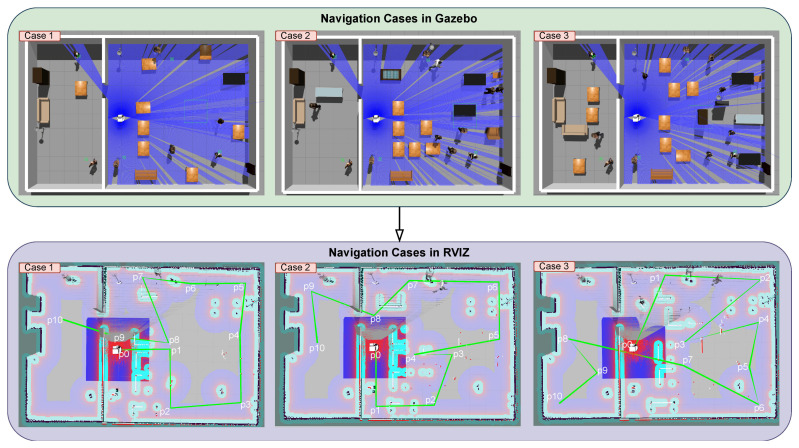
Illustration of three different scenarios simulated in Gazebo and visualized with Rviz to compare different navigation variants. Three different levels of navigation difficulty were simulated by adding objects to the environment: simple, moderate, and complex. The most used objects are desks (d), participants (p), and keyboards (k). The way-points are marked on the map as $P_{0}$ through $P_{10}$ and the sequence in the navigation task is symbolized by the green line.

The experiments were conducted in three different environments, each with increasing levels of difficulty: simple, moderate, and complex. These environments were designed in Gazebo to closely resemble real-world conditions, including both fixed and moving obstacles such as tables, chairs, and humans. In each environment, the robot navigated paths and plans defined by points $P_{0},P_{2},\ldots ,P_{10}$, marking the start and end points of path segments of segments as shown in Figure 8). These points were selected independently for each scenario. The purpose of these tests was to assess the robot’s navigation, obstacle avoidance, and goal completion in different scenarios. The results compared the performance of EGM and RTAB-Map across simple, moderate, and complex setups.

- Simple Environment: This setup has only a few obstacles, such as tables, keyboards, and sofas. The paths are wide and clear, making navigation easy for the robot. It provides a basic test of movement without major challenges.
- Moderate Environment: This setup adds more obstacles, like lamp holders and standing humans, to make navigation harder. The paths are narrower, requiring the robot to move carefully and adjust its route when needed. This helps test how well the robot can handle slightly more difficult spaces.
- Complex Environment: This setup is the most challenging. More obstacles are placed while the robot is moving, and the paths are made even narrower. This forces the robot to make precise movements and smart decisions to avoid collisions. It tests how well the robot adapts to unpredictable situations.

The navigation system was implemented using a Python script that processed data from /next_goal and move_base topics. The robot’s movement between waypoints was controlled using the cmd_vel topic. These topics are generally generated the in ROS navigation system.

### 4.2. Comparison Formulas Preparation

For simulation comparison, we assess the path coverage distance, time, battery efficiency, and CPU capabilities of the robot. The robot gathers odometry data from/mobile_base_controller/odom topic. To calculate the time interval between measurements, we subtract the previous timestamp $t_{i-1}$ from the current timestamp $t_{i}$, resulting in $\Delta t=t_{i}-t_{i-1}$. The linear velocity *v* is obtained from the absolute value of the *x*-component $v_{x}$ of the velocity reported in the odometry data. The incremental distance Δd is then computed as Δd=v·Δt. The total distance $D_{\mathrm{total}}$ is the sum of all incremental distances. The robot’s ability to reach the next goal is assessed by measuring its success in arriving at predetermined positions. The overall journey success rate is calculated based on the accumulated number of successfully reached points.

**Figure 9:**
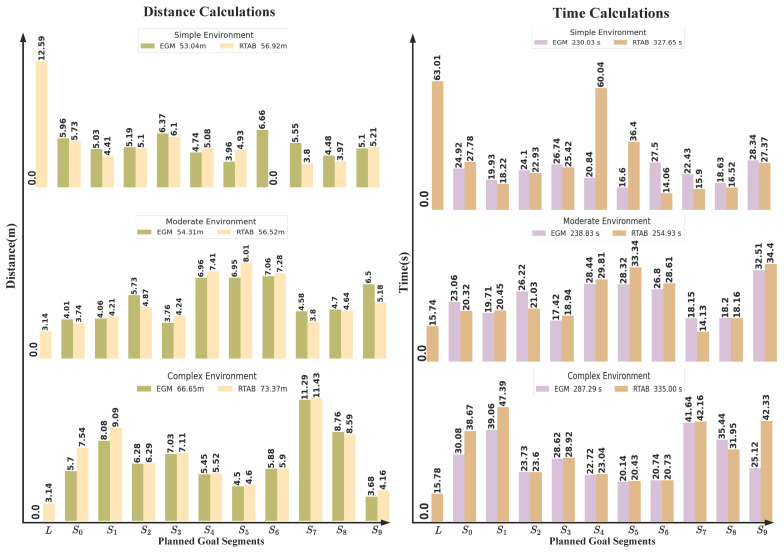
Showing robot performance parameters across three environments: simple, moderate, and complex. $S_{i}$ refers to the segment linking points sequentially, starting from the home position and extending up to point nine. The performance is shown between our EGM approach and RTAB-Map. The left column shows the distance calculations between points and their accumulated distance, while the right column shows the time between points and their accumulated time.

**Figure 10:**
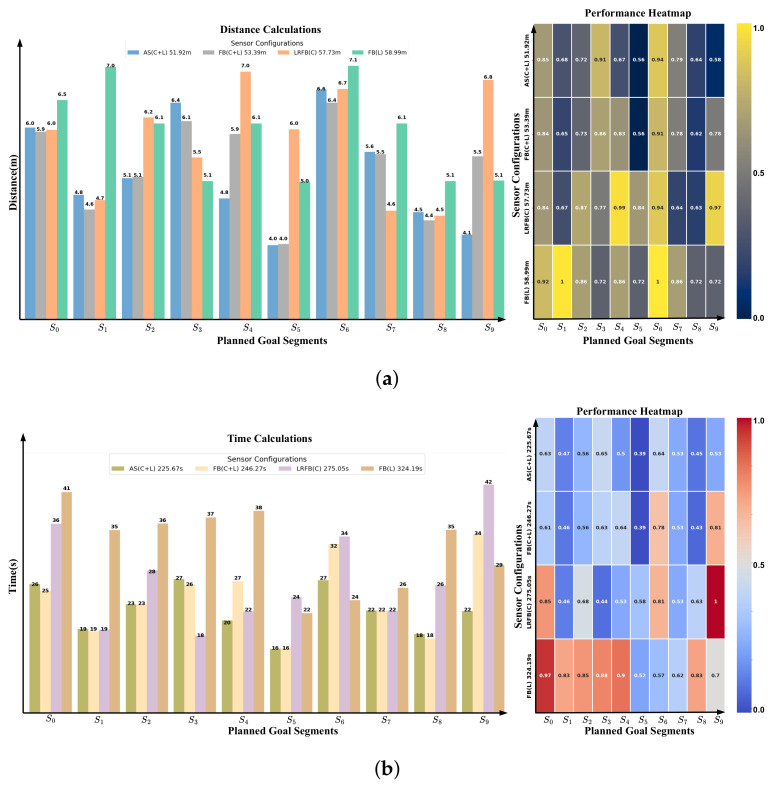
Performance comparison of the robot under different sensor configurations. The configurations include AS (C + L) (all sensors active: cameras and LiDAR), FB (C + L) (front and rear cameras plus LiDAR), LRFB (C) (cameras on all sides), and FB (L) (front and rear LiDAR sensors only). At (**a**) on the left shows the accumulated distance (in meters), with the right side displaying a heatmap illustrating the impact of combining LiDAR sensors with cameras on improving travel distance. At (**b**) on the left presents accumulated time (in seconds), while the right side features a heatmap showing the impact of combining LiDAR sensors with cameras on improving time efficiency.

For battery efficiency, we assume that the battery decreases by 0.01 units per distance step. The drop in battery percentage is calculated as ΔPercentage=Δd·0.01 and the drop in voltage is ΔVoltage=Δd·0.01. Finally, the results have been tabulated in Table 1, Table 2, Table 3 and Table 4. Also, they have been shown in Figure 9 and Figure 10 to be more understandable and clear for comparison.

### 4.3. Simulation

We used simulations to test our method and ensure it will be suitable for real-world applications. The simulations were conducted in Gazebo, a robot simulation environment, while Rviz was used for visualization. We tested our method in different scenarios to evaluate its ability to map and locate objects, comparing it with other SLAM methods, such as RTAB-Map.

#### 4.3.1. Rviz and Gazebo Object Projection

**Figure 11:**
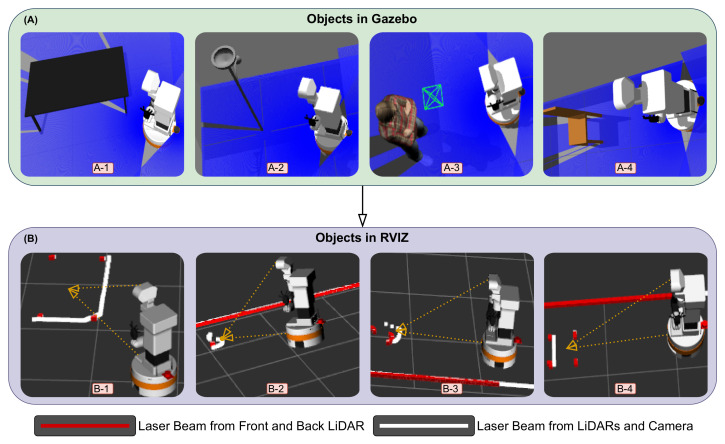
Comparison of object visualization in Gazebo and Rviz. (**A**) Simulation images from Gazebo. (**B**) Two-dimensional map representations in Rviz showing data from LiDAR (red lines) and cameras (white lines). The figure illustrates how the robot perceives different objects: (A-1, B-1) the desk boundaries, (A-2, B-2) the lamp holder’s head, (A-3, B-3) the human body, and (A-4, B-4) the chair boundaries.

To show the effectiveness of the fusion of the sensors that we analyzed, which are shown in Figure 1a,b, we demonstrate the effects using Gazebo and Rviz and the integration of the Python scripts that we prepared. Figure 11 demonstrates some of the fundamentals that we used in our study. Section A represents the objects and environment in Gazebo, and section B represents the visualization of the objects and their detection in Rviz. In this demonstration, we aimed to perform a comparison between the robot laser beam when it interacts with external objects while creating the map in the following scenarios: 1. when the robot just has its front LiDAR sensor, which will be represented as the red line; 2. when we integrate the robot back LiDAR sensor, which is also presented as a red line; and 3. when we merge all LiDAR sensors and depth cameras converting scan information, which is presented as a white line. The robot detects and interacts with various objects, which can be moving, fixed to the ground, or hanging from the ceiling. We selected four objects for our analysis.

Firstly, in images A-1 and B-1, the robot interacts with a table. When using only LiDAR data, the robot detects the table legs, shown by the red points. However, by combining LiDAR and camera data using our approach, all desk boundaries are detected and represented by white dots. For images A-2 and B-2, the robot interacts with a floor lamp that has a head at the top. Using only LiDAR data, the robot detects the vertical column of the lamp, represented by red dots. When camera data are integrated, the boundaries of the lamp head are also detected and projected as white dots, representing the combined sensor data. In images A-3 and B-3, the robot interacts with a human standing in front of it. With only LiDAR data, the robot detects the human legs, shown by red dots. When camera information is added, the robot detects the entire human body, projected as a 2D representation with a white line. Lastly, in images A-4 and B-4, the robot interacts with a chair. Using only LiDAR sensors, the robot detects the chair legs, represented by red dots. By integrating camera information, the boundaries of the chair are also detected, shown by white dots. These results highlight the advantages of fusing cameras with LiDAR sensors, enabling the robot to perceive its environment more effectively and capture finer details for improved navigation and interaction.

#### 4.3.2. Mapping and Localization

Localization and mapping are fundamental for any mobile robot to navigate efficiently and accurately within its environment [18,29]. It involves determining the robot’s position and orientation (pose) relative to a map of its surroundings. In the context of SLAM, mapping and localization are inherently interdependent; each relies on the other to function effectively [50]. A well-constructed map guides the robot through the environment, aiding in obstacle avoidance and path planning. In this section, we will discuss the methods used for map creation and localization, specifically focusing on three approaches: AMCL, Gmapping, and RTAB-Map. Each method offers unique advantages and is tailored to specific aspects of robot navigation.

A. Gmapping and AMCL

**Figure 12:**
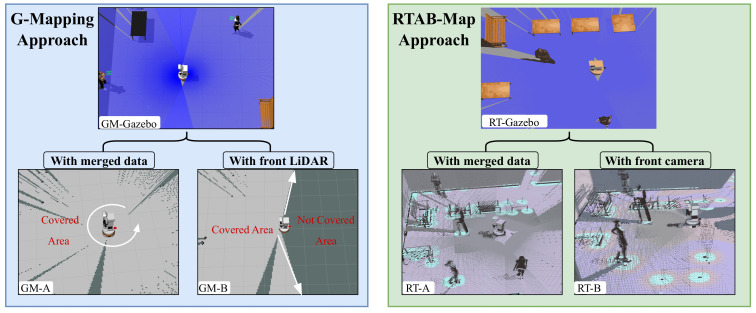
Comparison of mapping coverage in a Gazebo simulation environment with Rviz visualization, using different sensor setups. The **left image** blue box shows the Gmapping approach: GM-A covers the entire surroundings using two LiDAR sensors, while GM-B only covers the front area with a single LiDAR. The **right image** green box shows the RTAB-Map approach: RT-A uses all cameras for a full 360-degree view, and RT-B uses just the front camera and LiDAR, giving limited coverage.

Gmapping is a widely used ROS package for implementing SLAM. It utilizes scanning data from cameras and/or LiDAR sensors to map the environment where the robot operates. This package creates a 2D occupancy grid map from laser scan data while estimating the robot’s trajectory. The process involves grid mapping, data association, and pose graph optimization [20]. Furthermore, AMCL operates in a 2D space using a particle filter to track the robot’s pose relative to a known map. It publishes a transform between the /map and /odom frames, helping the robot understand its position within the environment. The particle filter estimates the robot’s (x,y,Θ) coordinates. Each particle represents a possible robot pose and has associated coordinates, orientation values, and weight based on the accuracy of its pose prediction. As the robot moves and acquires new sensor data, particles are re-sampled, retaining them with higher weights to refine the pose estimate [51]. Combining Gmapping with AMCL creates a more robust and reliable SLAM system. This combination improves both map creation time and accuracy. As shown in Figure 12, which is represented in the blue box, we have the same environment built in GM-Gazebo. By integrating additional sensors, such as back LiDAR and multiple RGB-D cameras as in GM-A, around 360 degrees are achieved while the robot is stationary.

However, it takes a longer time to move the robot to cover all places in the environment to create the map, as in GM-B. The setup reduces the time required to generate the map compared to using only front LiDAR and front cameras. The sensor setup accelerates mapping and improves detail, allowing the robot to detect elevated objects and obstacles more effectively. AMCL is better in fast localization compared to RTAB-Map localization as seen in Table 3 and Figure 9.

Finally, by optimizing the EGM and AMCL parameters to utilize data from all sensors, we achieve fast and accurate map generation, facilitating reliable localization and efficient navigation. It leverages the strengths of them, combined with sensor coverage, to develop the robot’s operational capabilities [52].

B. RTAB-Map

As mentioned before, RTAB-Map performs robot mapping and localization using advanced computer vision algorithms and loop closure detection logic [38]. This approach, while effective, results in high computational demands and longer localization times. During localization, the robot may experience delays in recognizing its position, which in turn affects its ability to reach its goal successfully. Additionally, creating a complete map requires the robot to travel and cover the entire environment, which can be time-consuming.

To address these challenges, we included extra cameras on the robot to speed up data matching and improve localization accuracy. As shown in Figure 12, represented in the green box, the RT-Gazebo environment consists of three participants and several tables. The RT-A display shows the map created using merged data from multiple cameras, while RT-B shows the map created using only the front camera sequentially. In the first scenario, RT-A, the three participants and the surrounding environment are detected easily and accurately. In contrast, in the second scenario, RT-B, where only one camera is used, only some features are detected initially, requiring the robot to move around the environment to detect the remaining features.

#### 4.3.3. SLAM and Navigation

In our experiment, the navigation setup was configured using the ROS Navigation Stack. First, the environment map was generated using the EGM package, which relies on laser rangefinder data. This map served as the foundation for robot localization. To localize the robot within this map, we used the AMCL package, which performs probabilistic localization using LiDAR data, the map, and initial pose estimates. We employed two mapping methods (Gmapping and RTAB-Map) and two localization techniques (AMCL and RTAB-Map loop closure detection). This allowed us to test the performance of the navigation stack under different conditions. Three distinct test cases were designed, see Figure 8, with varying environments to assess how the robot navigated under different scenarios.

The simulation was implemented and visualized using Gazebo and Rviz. Python 3.10 scripts were written to facilitate the simulation process and follow them synchronously. The environments were designed to allow for comparison across different simulation scenarios. Each case involved ten different goals, with the coordinates of each goal stored in TXT files. This was achieved using the /clicked_point topic published from the Rviz interface, allowing automatic storage of goal *x* and *y* coordinates for later use. Finally, a Python script published the *x* and *y* coordinates from the TXT file and visualized the path by connecting the goals to clearly display the planned trajectory.

#### 4.3.4. Path Planning

After setting up the simulation environment in Gazebo and configuring the /move_base parameters for all scenarios, the next phase of our experiment involved implementing path planning. Once the robot’s position and orientation are localized, a path must be planned to reach the target goal. Path planning is essential for autonomous navigation, ensuring that the robot can move from its current position to the destination while avoiding obstacles [19]. It is generally divided into two types: global path planning and local path planning.

1. Global Path Planning:

It involves finding an optimal or near-optimal path from the starting point to the destination, considering the entire environment. This planning is performed without considering the real-time constraints or dynamic obstacles in the environment [53]. Common algorithms used for global path planning include A* (A-star) and Dijkstra’s algorithm. These algorithms consider the entire map or environment, including obstacles, to determine the best path. In our setup, we implemented a global path planning algorithm using the ROS packages /global_planner and navfn. These packages generate a global path based on a static map of the environment. The /move_base package works in tandem with the global planner, handling navigation by sending velocity commands to the robot. This component collaborates with the global planner to manage navigation by sending velocity commands to the robot.

2. Local Path Planning:

Local path planning focuses on the robot’s immediate surroundings. It adjusts the robot’s trajectory in real time, helping it avoid dynamic obstacles while following the global path. Local path planners respond to changes in the environment, making necessary adjustments to avoid obstacles and maintain the planned trajectory. Techniques like potential fields, reactive methods, and artificial potential fields are commonly employed for this purpose [19]. We implemented local path planning using the /base_local_planner and /dwa_local_planner packages, allowing the robot to make real-time adjustments based on its local environment. However, we observed that the local planner sometimes failed in certain scenarios, especially when limited sensor data were available. This failure could be traced back to the global planner’s initial trajectory calculation. In situations where the robot approached obstacles, such as tables, the empty space between table legs could mislead the robot into thinking a passageway was available, causing collisions.

To ensure a fair comparison and consistent conditions across all three cases, we established ten fixed points on the maps, each with specific positions and orientations, as shown in Figure 8. These points were stored in TXT files using a Python script, ensuring uniformity in the robot’s path planning for each scenario. During the operation of the navigation system, the script continuously logged the positions of clicked points in Rviz to the TXT file by monitoring the /clicked_point topic. This standardization of initial positions, path trajectories, and /move_base parameters was essential for ensuring the repeatability and reproducibility of the experiments.

### 4.4. Implementation

Due to environmental constraints and the lack of specific sensors, we conducted a small-scale real-world implementation using the Tiago robot with pre-integrated sensors which are one RGB-D camera and one 2D LiDAR sensor. The experiment was set up using six independent segments forming a hexagonal path, initializing 1000 particles randomly; see Figure 6. The blue area indicates regions mapped with the original Gmapping technique, whereas the pink area represents our EGM approach, which integrates adaptive resampling and degeneracy handling into classical Gmapping.

This enhancement improved the distribution of particles, shown as red points, making them more concentrated and focused, thus allowing the robot to obtain more precise localization and estimation of its position. The green, black, and blue lines in the visualization represent the planned path, the estimated path using original Gmapping, and the estimated path using our improved method, respectively. The alignment differences between these paths highlight the improvements in robot position estimation.

The quantitative results, summarized in Table 5, illustrate key performance metrics:

- Traveled Distance: Using our EGM, the total traveled distance was 14.95 m, compared to 16.10 m with the original GM. This reduction demonstrates improved estimation of the robot position based on the distributed particles and in collaboration with the obtained sensor fused information.
- Time Required: The journey with EGM was completed in 64.70 s, whereas GM required 70.90 s.
- Goal Achievement: Both methods successfully reached all goal points (100% success rate), confirming that EGM and classical GM are scoring the same target for goal achievement.
- Overlap Ratio: The overall overlap ratio with EGM was 68%, while GM had 33.7%. The high overlap ratio in EGM suggests that the particles were more focused, reducing unnecessary dispersion and enhancing the robot’s precise positioning.
- Average Error: The cumulative localization error in EGM was 0.85 m, while GM had a higher error of 1.56 m. This 45.5% reduction in error demonstrates the effectiveness of adaptive resampling in maintaining accurate position estimates.

The overlap ratio was used to evaluate the alignment of estimated positions with the true robot position by considering the fraction of particles within a threshold distance of 0.5 m. It was computed as follows:

(11) $$d_{i}=\sqrt{{\left(x_{i}-x_{\mathrm{true}}\right)}^{2}+{\left(y_{i}-y_{\mathrm{true}}\right)}^{2}}$$

where $d_{i}$ is the Euclidean distance between particle $\left(x_{i},y_{i}\right)$ and the true position $\left(x_{\mathrm{true}},y_{\mathrm{true}}\right)$. The number of particles satisfying $d_{i}<0.5$ m was then used to compute the overlap ratio:

(12) $$\mathrm{Overlap}\;\mathrm{Ratio}=\frac{\mathrm{Number}\;\mathrm{of}\;\mathrm{particles}\;\mathrm{within}\;\mathrm{threshold}}{\mathrm{Total}\;\mathrm{number}\;\mathrm{of}\;\mathrm{particles}}$$

These findings emphasize that integrating adaptive resampling and degeneracy handling in Gmapping enhances particle consistency, leading to more realistic and efficient mapping. The reduction in traveled distance, time, and localization error while maintaining a high goal success rate proves the effectiveness of our approach in real-world implementations.

## 5. Results and Discussion

This section presents the evaluation and discussion of our proposed method, focusing on multi-sensor integration with optimal fusion strategies and enhanced mapping.

### 5.1. Evaluation Metrics

To assess the robot’s performance, we considered key metrics that evaluate its efficiency and effectiveness in navigation. Task accomplishment was measured by the robot’s ability to successfully complete assigned tasks. Time and distance efficiency were tracked by recording the time and distance between goals to evaluate navigation performance and compute the total accumulated time and distance. Success rates were analyzed by assessing the percentage of goals successfully reached versus failures due to obstacles, providing insight into the robot’s obstacle avoidance capabilities (see Table 1 and Table 3 and Figure 9 and Figure 10). Resource consumption, including battery efficiency and CPU usage, was monitored to understand the computational and energy demands during operation. Stability was evaluated by analyzing the robot’s linear and angular velocities to ensure smooth and consistent movement (see Table 2 and Table 4). To further analyze time and distance efficiency, we normalized execution times and distances relative to the maximum recorded time and distances. Then, we visualized them using a heatmap. Warmer colors with higher numbers indicate longer execution times and longer distances, while cooler colors with small numbers represent faster performance, allowing for an intuitive comparison of sensor configurations.

For real-world implementation, as described in Section 4.4, we followed the same strategy as in the simulation while additionally incorporating the overlap ratio metric, which quantifies the alignment of the estimated robot position relative to the given position.

### 5.2. Sensor Configuration Analysis

To evaluate the impact of different sensor configurations on navigation performance, we conducted experiments with four distinct setups using our EGM method:

1. AS (C + L): All sensors, incorporating both cameras and LiDARs.
2. FB (C + L): Front and back cameras and LiDARs.
3. LRFB (C): Just left, right, front, and back cameras.
4. FB (L): Just front and back LiDAR sensors.

As shown in Figure 10 and Table 1 and Table 2, all the details and comparisons discussed in this section are provided in these visualizations and tables. Utilizing all available sensors, AS (C + L), which combines all cameras and LiDARs, significantly improved navigation efficiency. This configuration resulted in the shortest travel distance of 51.8 m and the lowest travel time of 223.7 s, outperforming all other setups.

Incorporating only front and back cameras and LiDARs, FB (C + L), enabled the robot to reach all goals successfully. However, it required more time 54.3 s and travel distance 245.5 m compared to AS (C + L). When relying solely on cameras, LRFB (C), the robot became stuck at one goal, preventing full completion of the planned journey. In contrast, using only LiDAR sensors, FB (L), resulted in just 40% navigation success, with the robot failing to complete the journey due to obstacles. Moreover, the heatmap indicates that areas with shorter time and distance values are concentrated at the top of the grid. As sensor configurations are reduced, both execution time and distance increase, visually demonstrating the performance decline with fewer sensors incorporated. Battery and voltage consumption were minimized using AS (C + L), with a charge decrease of 5.3% and a voltage drop of 2.6%. CPU usage was also the lowest in this configuration. Stability analysis further demonstrated that AS (C + L) provided more consistent linear and angular velocity, ensuring smoother motion.

In real-time implementation, as shown in Table 5, a slight improvement was observed when comparing EGM with GM in terms of time, distance traveled, overlap ratio between planned and estimated segments, and average error generated. Although environmental constraints limited the scale of our experiments compared to the simulations, the results were still sufficient to demonstrate that incorporating both visual and LiDAR-based data, combined with a well-designed fusion framework and adaptive particle assembly, allows the robot to navigate effectively in complex environments in both simulation and real-world applications.

### 5.3. Environmental Configuration Analysis

This section evaluates the performance of the proposed EGM method across different environments: simple, moderate, and complex. The goal is to fairly compare the impact of adaptive sampling and degeneracy handling, combined with our sensor fusion technique, against the RTAB-Map approach in varying environmental conditions. The evaluation was conducted using various performance metrics, as shown in Figure 9 and Table 3 and Table 4.

In a simple environment, the proposed EGM method completed the task in 230.04 s, compared to 327.65 s using the RTAB method. The distance covered was 53.04 m with our approach versus 56.92 m with RTAB. Additionally, the RTAB-Map approach caused the robot to get stuck at two goals, while our method successfully reached all goals.

In a moderate environment, the proposed method completed the task in 238.83 s, compared to 254.93 s with the RTAB method. The distance covered was 54.31 m with our approach versus 58.27 m with RTAB. Similarly, the RTAB-Map approach caused the robot to get stuck at one goal, whereas our method successfully navigated to all goals.

In a complex environment, the proposed method completed the task in 287.29 s, compared to 335.0 s with the RTAB method. The distance covered was 66.65 m with our approach versus 73.37 m with RTAB. Again, the RTAB-Map approach resulted in the robot getting stuck at two goals, while our method successfully completed the entire task. Additional metrics, including charge and voltage consumption, CPU usage, and motion stability, further highlight the advantages of our approach. While both methods showed increased CPU demand as environmental complexity grew, our approach maintained lower CPU usage in certain cases. In terms of motion stability, both methods demonstrated stable linear and angular velocities, ensuring smoother navigation.

These results demonstrate that the proposed method has slightly competitive results against RTAB in terms of time efficiency and distance traveled across various environments. This is primarily due to the localization requirements of RTAB-Map, which involves processing intense features to help the robot localize and move to the next goal.

## 6. Conclusions

In this work, we developed an improved SLAM system for mobile robot navigation by combining our sensor fusion technique with an enhanced mapping approach. Our fusion method converts RGB-D point clouds into 2D laser scans, allowing seamless integration with LiDAR data, while parallel noise filtering improves sensor reliability. To enhance localization, we introduced Enhanced Gmapping (EGM), which includes adaptive resampling and degeneracy handling to maintain particle diversity and degeneracy handling to prevent mapping errors. These improvements made the system more stable and accurate. Our approach slightly improved traditional methods, achieving 8% less traveled distance, 13% faster processing, and 15% better goal completion in simulations. In real-world tests, EGM further reduced the traveled distance by 3% and execution time by 9%, demonstrating its effectiveness. Despite these improvements, challenges remain, such as difficulty detecting objects with gaps, like table legs, which can cause navigation errors. To address this, we plan to integrate AI-based solutions, including LSTM for better memory of visited locations and CNNs for improved object recognition. Additionally, we will test the system in more complex and dynamic environments beyond controlled simulations to further validate its real-world applicability. These advancements will improve the system’s adaptability and reliability, making it more effective for real-world autonomous navigation.

## Figures and Tables

**Table 1:** Different sensor configurations’ goal achievements.

Sensor Configurations	AS (C + L)			FB (C + L)			LRFB (C)			FB (L)		
Argument Parameter	D (m)	T (s)	G	D (m)	T (s)	G	D (m)	T (s)	Goal	D (m)	T (s)	G
*S*_0_	6.0	26.6	✓	5.9	25.5	✓	5.9	36.0	✓	6.5	41.0	✓
*S*_1_	4.8	19.6	✓	4.6	19.2	✓	4.7	19.4	✓	7.0	35.2	✗
*S*_2_	5.1	23.5	✓	5.1	23.4	✓	6.2	28.7	✓	6.1	36.3	✓
*S*_3_	6.4	27.0	✓	6.1	26.5	✓	5.5	18.9	✓	5.1	37.2	✗
*S*_4_	4.8	20.3	✓	5.9	27.2	✓	7.3	22.3	✓	6.1	38.1	✗
*S*_5_	4.0	16.5	✓	3.9	16.4	✓	3.9	24.3	✓	5.0	22.2	✓
*S*_6_	6.5	27.2	✓	7.4	32.2	✓	6.6	34.3	✓	7.1	24.2	✗
*S*_7_	5.6	22.3	✓	5.5	22.4	✓	4.6	22.4	✓	6.1	26.3	✗
*S*_8_	4.5	18.3	✓	5.1	18.4	✓	4.5	26.4	✗	5.1	35.1	✓
*S*_9_	4.1	22.4	✓	5.5	34.3	✓	6.8	42.3	✓	5.1	29.3	✗
**Total, Avg**	**51.8**	**223.7**	**100%**	**54.3**	**245.5**	**100%**	**56.0**	**275.0**	**90%**	**59.2**	**325.0**	**40%**

Showing comparison of navigation results of four different sensor configurations in the same environment (see Figure 10). AS (C + L) represents all sensors on the robot; FB (C + L) represents front and rear cameras and LiDAR sensors; LRFB (C) represents left, right, rear, and front cameras; and FB (L) represents rear and front LiDAR only. S_i_ refers to the segment linking points sequentially, starting from the home position and extending up to point nine.

**Table 2:** Different sensor configurations’ work efficiency.

Approach	AS (C + L)	FB (C + L)	LRFB (C)	FB (L)
Parameter				
**Charge Decrease %**	**5.3**	5.5	4.67	6.2
Voltage Decrease %	**2.6**	2.8	2.3	2.2
CPU Usage %	**0.01**	0.09	0.19	0.57
Average Linear Velocity m/s	0.15, **Stable**	0.15, **Stable**	0.15, Not Stable	0.15, Not Stable
Average Angular Velocity rad/s	0.5, **Stable**	0.5, **Stable**	0.5, Not stable	0.5, Not Stable

Showing the robot’s efficiency across four sensor setups: AS (C + L) (all sensors), FB (C + L) (front/back cameras + LiDAR), LRFB (C) (all cameras), and FB (L) (front/back LiDAR). It shows battery and voltage decreases, CPU usage, and stability of movement. AS (C + L) provided stable movement with moderate battery use, while FB (C + L) caused the most battery drain and instability.

**Table 3:** Environmental navigation goal achievements.

Environments	Simple Environment						Moderate Environment						Complex Environment					
Arguments	D (m)		T (s)		Goal.A		D (m)		T (s)		Goal.A		D (m)		T (s)		Goal.A	
	EGM	RTAB	EGM	RTAB	EGM	RTAB	EGM	RTAB	EGM	RTAB	EGM	RTAB	EGM	RTAB	EGM	RTAB	EGM	RTAB
Localization (L)	0.00	12.59	0.00	63.01	-	-	0.00	3.14	0.00	15.74	-	-	0.00	3.14	0.00	15.78	-	-
*S*_0_	5.96	5.73	24.92	27.78	✓	✓	4.01	3.74	23.06	20.32	✓	✓	5.70	7.54	30.08	38.67	✓	✓
*S*_1_	5.03	4.41	19.93	18.22	✓	✓	4.06	4.21	19.71	20.45	✓	✓	8.08	9.09	39.06	47.39	✓	✓
*S*_2_	5.19	5.10	24.10	22.93	✓	✓	5.73	4.87	26.22	21.03	✓	✓	6.20	6.29	23.73	23.60	✓	✓
*S*_3_	6.37	6.10	26.74	25.42	✓	✓	3.76	4.24	17.42	18.94	✓	✓	7.03	7.11	28.62	28.92	✓	✓
*S*_4_	4.74	5.08	20.84	60.04	✓	✗	6.96	7.41	28.44	29.81	✓	✓	5.45	5.52	22.72	23.04	✓	✗
*S*_5_	3.96	4.93	16.60	36.40	✓	✓	6.95	8.01	28.32	33.34	✓	✗	4.50	4.60	20.14	20.43	✓	✓
*S*_6_	6.66	0.00	27.50	14.06	✓	✗	7.06	7.28	26.80	28.61	✓	✓	5.88	5.90	20.74	20.73	✓	✓
*S*_7_	5.55	3.80	22.43	15.90	✓	✓	4.58	3.80	18.15	14.13	✓	✓	11.29	11.43	41.64	42.16	✓	✓
*S*_8_	4.48	3.97	18.63	16.52	✓	✓	4.70	4.64	18.20	18.16	✓	✓	8.76	8.59	35.44	31.95	✓	✗
*S*_9_	5.10	5.21	28.34	27.37	✓	✓	6.50	5.18	32.51	34.40	✓	✓	3.68	4.16	25.12	42.33	✓	✓
**Total**	**53.04**	**56.92**	**230.04**	**327.65**	**100%**	**80%**	**54.31**	**56.52**	**238.83**	**254.93**	**100%**	**90%**	**66.65**	**73.37**	**287.29**	**335.00**	**100%**	**80%**

Environmental navigation goal achievements: Distance, time, and goal achievement for different environment setups: simple, moderate, complex. Si refers to the robot’s direction from goal to goal. EGM and RTAB refer to using Enhanced Gmapping and RTAB-Map, respectively. D(m) represents the distance in meters, T(s) represents the time in seconds, and Goal. A indicates the goal achievement. For visual details, see Figure 9.

**Table 4:** Environmental configuration work efficiency.

Environments	Simple Environment		Moderate Environment		Complex Environment	
Arguments	**EGM**	**RTAB**	**EGM**	**RTAB**	**EGM**	**RTAB**
Charge Decrease %	5.31	**4.43**	5.43	**5.34**	**6.66**	7.02
Voltage Decrease %	2.65	**2.22**	2.72	**2.67**	**3.33**	3.51
CPU Usage %	**0.04**	0.14	**0.4**	0.84	**0.55**	0.92
Average Linear Velocity m/s	0.15, Stable	0.15, Stable	0.15, Stable	0.15, Stable	0.15, Stable	0.15, Not Stable
Angular Velocity rad/s	0.5, Stable	0.5, Not Stable	0.5, Stable	0.5, Stable	0.5, Stable	0.5, Stable

Showing the robot’s efficiency across EGM and RTAB-Map methods. Showed slight improvements across most cases, maintaining stable linear and angular velocities and less voltage and battery consumption compared to RTAB-Map.

**Table 5:** Real-world implementation table.

Segments (m)	Traveled Distance (m)		Time (s)		Goals Achievement		Overlap Ratio (%)		Average Error (m)	
	EGM	GM	EGM	GM	EGM	GM	EGM	GM	EGM	GM
$P_{0}\to P_{1}$, 2.0 m	2.10	2.25	8.80	9.50	**✓**	**✓**	48.00	35.00	0.05	0.06
$P_{1}\to P_{2}$, 2.5 m	2.65	2.80	11.10	12.61	**✓**	**✓**	68.00	33.00	0.15	0.30
$P_{2}\to P_{3}$, 1.0 m	1.10	1.25	5.20	6.5	**✓**	**✓**	67.00	36.00	0.10	0.20
$P_{3}\to P_{4}$, 1.5 m	1.65	1.75	9.85	10.55	**✓**	**✓**	71.00	32.00	0.15	0.35
$P_{4}\to P_{5}$, 3.2 m	3.45	3.55	14.55	15.37	**✓**	**✓**	75.00	32.00	0.25	0.37
$P_{5}\to P_{0}$, 3.8 m	4.00	4.50	15.20	16.37	**✓**	**✓**	78.00	34.00	0.15	0.28
**Total-Avg, 14.0**	**14.95**	**16.10**	**64.70**	**70.90**	**100%**	**100%**	**68.00**	**33.70**	**0.85**	**1.56**

Real-time performance comparison between Enhanced Gmapping (EGM) and Gmapping (GM) for the robot’s path from goal P_*i*_ to P_*i+1*_, including traveled distance, time, goal achievement, overlap ratio, and average error.

## Data Availability

The original contributions presented in this study are included in the article. Further inquiries can be directed to the corresponding authors.
